# Supplementary material for: Re-expression of SMARCA4/BRG1 in small cell carcinoma of ovary, hypercalcemic type (SCCOHT) promotes an epithelial-like gene signature through an AP-1-dependent mechanism
Source: eLife. 2020 Dec 23;9:e59073. doi: 10.7554/eLife.59073 (PMC7813545; doi:10.7554/eLife.59073)
Supplement: Transparent reporting form [file elife-59073-transrepform.docx]

***eLife’s* transparent reporting form**

We encourage authors to provide detailed information *within their submission* to facilitate the interpretation and replication of experiments. Authors can upload supporting documentation to indicate the use of appropriate reporting guidelines for health-related research (see [EQUATOR Network](http://www.equator-network.org/%20)), life science research (see the [BioSharing Information Resource](https://biosharing.org/)), or the [ARRIVE guidelines](http://www.plosbiology.org/article/info:doi/10.1371/journal.pbio.1000412) for reporting work involving animal research. Where applicable, authors should refer to any relevant reporting standards documents in this form.

If you have any questions, please consult our Journal Policies and/or contact us: [editorial@elifesciences.org](mailto:editorial@elifesciences.org).

**Sample-size estimation**

- You should state whether an appropriate sample size was computed when the study was being designed
- You should state the statistical method of sample size computation and any required assumptions
- If no explicit power analysis was used, you should describe how you decided what sample (replicate) size (number) to use

Please outline where this information can be found within the submission (e.g., sections or figure legends), or explain why this information doesn’t apply to your submission:

Sample-size estimations were not used in this manuscript. We used the number of replicates commonly used in these types of analyses. For Parental RNA-seq and ATAC-seq studies we increased the number of replicates to 4 biological replicates.

**Replicates**

- You should report how often each experiment was performed
- You should include a definition of biological versus technical replication
- The data obtained should be provided and sufficient information should be provided to indicate the number of independent biological and/or technical replicates
- If you encountered any outliers, you should describe how these were handled
- Criteria for exclusion/inclusion of data should be clearly stated
- High-throughput sequence data should be uploaded before submission, with a private link for reviewers provided (these are available from both GEO and ArrayExpress)

Please outline where this information can be found within the submission (e.g., sections or figure legends), or explain why this information doesn’t apply to your submission:

Biological replicate defined as: Distinct, separate experiments performed by different passages of cells with experiments performed on different days.

Technical replicate defined as: Additional assay performed with the same set of samples.

Parental BIN67 RNA-seq, ATAC-seq = 4 biological replicates per condition

A427, COV434, and SCCOHT-1 RNA-seq = 3 biological replicates per condition

Proteomics = 3 biological replicates per condition

CUT&RUN = 2 biological replicates per condition

AFOS RNA-seq = 2 biological replicates per condition

WB on Figure 6 = representative western blot (from at least 3+ biological replicates) as a technical replicate of samples to show all proteins that migrate at similar molecular weights.

WB in Fig1_S1: Two biological replicates of transfected samples in each cell line with the western blot performed in technical duplicates. One of the technical replicates is shown in the figure.

Replicates are additionally indicated in the material and methods and within figure legends.

Raw fastq files and processed data for ATAC-seq, RNA-seq, and CUT&RUN-seq studies have been deposited in Gene Expression Omnibus (GEO) database with the accession number: GSE151026.

Proteomics data was deposited in PRIDE database (accession #PXD014134).

For all data deposited, a reviewer access link has been provided to the editors to distribute to reviewers.

**Statistical reporting**

- Statistical analysis methods should be described and justified
- Raw data should be presented in figures whenever informative to do so (typically when N per group is less than 10)
- For each experiment, you should identify the statistical tests used, exact values of N, definitions of center, methods of multiple test correction, and dispersion and precision measures (e.g., mean, median, SD, SEM, confidence intervals; and, for the major substantive results, a measure of effect size (e.g., Pearson's r, Cohen's d)
- Report exact p-values wherever possible alongside the summary statistics and 95% confidence intervals. These should be reported for all key questions and not only when the p-value is less than 0.05.

Please outline where this information can be found within the submission (e.g., sections or figure legends), or explain why this information doesn’t apply to your submission:

Statistical reporting is indicated in the figure legend, main text, and stated in material and methods. P-values are indicated within the main text, in figure legends, and within figures.

(For large datasets, or papers with a very large number of statistical tests, you may upload a single table file with tests, Ns, etc., with reference to sections in the manuscript.)

**Group allocation**

- Indicate how samples were allocated into experimental groups (in the case of clinical studies, please specify allocation to treatment method); if randomization was used, please also state if restricted randomization was applied
- Indicate if masking was used during group allocation, data collection and/or data analysis

Please outline where this information can be found within the submission (e.g., sections or figure legends), or explain why this information doesn’t apply to your submission:

The sample size (n = # of genes or peaks) is indicated within each figure, in the figure legends, and in the main text.

Randomization was not applicable to the design of these experiments, parental cell lines were split into two and transfected with various constructs in each biological replicate independently.

**Additional data files (“source data”)**

- We encourage you to upload relevant additional data files, such as numerical data that are represented as a graph in a figure, or as a summary table
- Where provided, these should be in the most useful format, and they can be uploaded as “Source data” files linked to a main figure or table
- Include model definition files including the full list of parameters used
- Include code used for data analysis (e.g., R, MatLab)
- Avoid stating that data files are “available upon request”

Please indicate the figures or tables for which source data files have been provided:

Additional files have been included as supplemental tables that have the complete RNA-seq and proteomics results for all datasets. Data underlying the scatterplots for Transcription Factor Motif analysis are provided as supplemental table 2 and supplemental table 7. Location of ATAC-seq peaks used for plotting are found Supplemental Table 5. All CUT&RUN peaks from each condition are found in Supplemental Figure 6

Parameters used for data analysis, including program and version numbers, have been indicated in the material and methods.

Processed data (bigwig files, peak calls, and raw gene counts) have been deposited in GEO.
